# *Notes from the Field:* Multipathogen Respiratory Virus Testing Among Primary and Secondary School Students and Staff Members in a Large Metropolitan School District — Missouri, November 2, 2022–April 19, 2023

**DOI:** 10.15585/mmwr.mm7228a4

**Published:** 2023-07-14

**Authors:** Jennifer L. Goldman, Brian R. Lee, Janelle Porter, Anila Deliu, Shannon Tilsworth, Olivia M. Almendares, Sadia Sleweon, Hannah L. Kirking, Rangaraj Selvarangan, Jennifer E. Schuster

**Affiliations:** ^1^Children’s Mercy Kansas City, Kansas City, Missouri; ^2^North Kansas City School District, Kansas City, Missouri; ^3^Coronavirus and Other Respiratory Viruses Division, National Center for Immunization and Respiratory Diseases, CDC.

Respiratory virus infections are common in school-aged children (*1*). Although children spend most of their awake hours in the school setting, few data are available on the prevalence of respiratory viruses in schools. Surveillance for respiratory viruses other than SARS-CoV-2 has not been widely conducted in primary and secondary schools (*2*).

## Prospective Surveillance and Preliminary Results

To determine the prevalence of respiratory viruses in school students and staff members, prospective surveillance was implemented in a large metropolitan school district in Kansas City, Missouri with 33 pre-Kindergarten (pre-K)–grade 12 schools during the 2022–23 school year. All district students and staff members were eligible to enroll in opt-in respiratory virus testing and symptom surveys irrespective of the presence of symptoms; enrollment information was sent by the school district using existing communication channels. Self-collected anterior nasal swabs were obtained monthly and tested using multiplex viral polymerase chain reaction.* Thirty-six hours before each scheduled monthly test, an electronic survey was sent to enrolled participants (or their parent or guardian) inquiring about respiratory virus infection symptoms during the preceding 7 days.^†^ Logistic regression models were used to compare positivity across age groups. Regression models accounted for clustering within schools when calculating cluster-robust SEs. Percentile-based bootstrapped CIs were calculated using Stata 17 software (version 17.0; StataCorp). The goal of this report is to share timely virus testing results during ongoing surveillance. This activity was reviewed by CDC and was conducted consistent with applicable federal law and CDC policy.^§^

Among the 894 total participants, 639 (71.5%) were students (representing 3.0% of total district enrollment of 21,419), and 255 (28.5%) were staff members (representing 7.1% of the total 3,577 district full-time staff members). Demographic characteristics of participants were similar to those reported districtwide, except that the proportion of female participants was higher (60.7%) than that from districtwide estimates (51.1%), and the proportion of students qualifying for free or reduced price meals was lower (31.3% versus 38.0%)^¶^ (*3*). Among students, the median age was 10.1 years (IQR = 7.5–12.5 years), 289 (45.2%) were male, 406 (63.5%) were non-Hispanic White (White), 80 (12.5%) were Hispanic or Latino (Hispanic), 49 (7.7%) were non-Hispanic multiracial (multiracial), and 46 (7.2%) were non-Hispanic Black or African American (Black). Among staff members, the median age was 42.2 years (IQR = 34.3–51.1 years), 21 (8.2%) were male, 214 (83.9%) were White, 12 (4.7%) were Hispanic, seven (2.7%) were multiracial, and five (2.0%) were Black.

A total of 3,232 surveillance specimens were tested, including 872 (27.0%) from staff members and 2,360 (73.0%) from students (Table). Student specimens included 90 (2.8%) from pre-K students, 1,413 (43.7%) from elementary school students, 479 (14.8%) from middle school students, and 378 (11.7%) from high school students. A median of four specimens per participant (IQR = 3–5) were collected; these included 80 (2.5%) in November, 404 (12.5%) in December, 711 (22.0%) in January, 798 (24.7%) in February, 824 (25.5%) in March, and 415 (12.83%) in April. Overall, 805 (24.9%) specimens tested positive for any virus (95% CI = 23.4%–26.4%). A substantially higher percentage of pre-K specimens tested positive (40.0%) compared with staff member specimens (14.1%) (p<0.001).** Overall, rhinovirus/enterovirus (RV/EV) was detected most frequently (392; 12.1%), followed by all seasonal coronaviruses including NL63, HKU1, OC43, and 229E (181; 5.6%). Among specimens from pre-K and elementary school students, RV/EV (14.4% and 17.1%, respectively), adenovirus (12.2% and 3.3%, respectively), seasonal coronaviruses (6.7% and 8.1%, respectively) and human metapneumovirus (4.4% and 3.7%, respectively) were frequently detected. Among staff member specimens, RV/EV (4.8%), seasonal coronaviruses (3.8%), and SARS-CoV-2 (3.3%) were frequently detected. Influenza and respiratory syncytial virus (RSV) were infrequently detected from surveillance specimens, possibly because testing commenced after the occurrence of early seasonal peaks (*4*,*5*). More than one virus was detected in 81 (2.5%) specimens.

**TABLE:** Multiplex polymerase chain reaction testing results from self-collected nasal swabs from students and staff members participating in prospective respiratory virus surveillance testing in schools (N = 3,232) — Missouri, November 2, 2022–April 19, 2023

Virus detected, no. (%)	School level, no. of specimens (% of total)									
	Pre-K n = 90 (2.8)		Elementary school n = 1,413 (43.7)		Middle school n = 479 (14.8)		High school n = 378 (11.7)		Staff members n = 872 (27.0)	
	No.	% (95% CI)	No.	% (95% CI)	No.	% (95% CI)	No.	% (95% CI)	No.	% (95% CI)
Any virus detection, 805 (24.9)*	**36**	**40.0 (26.4–45.2)**	**466**	**33.0 (29.6–36.7)**	**117**	**24.4 (18.1–29.2)**	**63**	**16.7 (12.9–20.5)**	**123**	**14.1 (10.9–16.5)**
Rhinovirus/Enterovirus, 392 (12.1)	13	14.4 (3.2–29.8)	241	17.1 (14.5–20.0)	65	13.6 (7.9–17.6)	31	8.2 (6.0–10.3)	42	4.8 (3.2–6.5)
Adenovirus, 70 (2.2)	11	12.2 (7.6–21.0)	46	3.3 (2.2–4.5)	7	1.5 (0.8–3.8)	3	0.8 (0.4–2.5)	3	0.3 (0.1–0.8)
Seasonal coronavirus, 181 (5.6)	6	6.7 (2.9–8.8)	114	8.1 (6.7–9.3)	17	3.5 (1.1–5.8)	11	2.9 (1.2–4.9)	33	3.8 (2.7–5.0)
Human metapneumovirus, 93 (2.9)	4	4.4 (2.4–11.9)	52	3.7 (2.6–5.0)	13	2.7 (1.0–4.2)	7	1.9 (1.2–3.2)	17	1.9 (0.9–3.2)
SARS-CoV-2, 77 (2.4)	2	2.2 (1.5–4.2)	29	2.1 (1.3–2.9)	9	1.9 (0.7–3.8)	8	2.1 (0.7–4.8)	29	3.3 (2.0–5.2)
Parainfluenza virus, 29 (0.9)	2	2.2 (1.0–8.5)	13	0.9 (0.5–1.3)	5	1.0 (0.3–2.5)	4	1.1 (0.6–3.3)	5	0.6 (0.2–1.3)
RSV,^✝^ 23 (0.7)	1	1.1 (0.6–2.8)	11	0.8 (0.3–1.4)	5	1.0 (0.5–2.8)	4	1.1 (0.3–3.0)	2	0.2 (0.1–0.6)
Influenza A,^†^ 21 (0.6)	0	—	11	0.8 (0.4–1.2)	5	1.0 (0.4–2.5)	2	0.5 (0.2–1.1)	3	0.3 (0.1–0.7)
Influenza B, 2 (0.1)	0	—	0	—	1	0.2 (0.1–1.2)	0	—	1	0.1 (0.1–0.1)
**Reported symptoms during previous 7 days, no.**										
Asymptomatic, 1,628	28	31.1 (18.0–41.6)	657	46.5 (42.0–51.2)	246	51.4 (37.8–57.2)	220	58.2 (46.9–65.4)	477	54.7 (49.0–60.7)
One or more symptoms, 765	37	41.1 (32.3–65.0)	343	24.3 (21.3–27.5)	111	23.2 (18.0–33.6)	53	14.0 (6.8–18.6)	221	25.3 (21.3–29.6)
Survey not completed, 839	25	27.8 (3.1–40.2)	413	29.2 (24.1–34.1)	122	25.5 (18.6–36.4)	105	27.8 (19.9–45.8)	174	20.0 (15.5–24.6)

**Abbreviations:** Pre-K = pre-Kindergarten; RSV = respiratory syncytial virus.

* Viral detections are not mutually exclusive.

^✝^ RSV peak occurred during October–November 2022; influenza peak occurred during October–December 2022. https://www.cdc.gov/surveillance/resp-net/dashboard.html (Accessed July 6, 2023).

Among the 3,232 symptom surveys sent, 2,393 (74.0%) were completed. Pre-K students had the highest prevalence of reporting one or more symptoms (41.1%) compared with high school students, among whom prevalence of symptoms was lowest (14.0%) (p<0.001).

## Preliminary Conclusions

The findings in this report are subject to at least three limitations. First, participation in this program is voluntary; participants who opt in might not be representative of the full school population. Second, all nasal swabs were collected by participants, and approximately 25% of specimens did not have known symptomatology because of lack of survey response. Finally, this early report describes positive laboratory results, not the likelihood of individual students or staff members receiving a positive test result during the school year.

The COVID-19 pandemic highlighted the gap in knowledge related to the prevalence and symptoms of respiratory viruses among children and in schools. These data are important to improve understanding of the epidemiology of respiratory viruses in a school setting, including but not limited to SARS-CoV-2. To support healthy learning environments for all, it is important to implement strategies to prevent and reduce the spread of infectious diseases, including staying up to date with recommended vaccinations, including COVID-19 and influenza vaccines, practicing good hand hygiene and respiratory etiquette, staying home when sick, and improving indoor ventilation. Final results of this surveillance effort will assist in refining the spectrum of panrespiratory approaches to respiratory virus prevention and could direct guidance in primary and secondary schools.

## Footnotes

* Testing was performed using Hologic Panther Fusion Assays for adenovirus; human metapneumovirus; influenza A and B viruses; parainfluenza virus, types 1–4; rhinovirus/enterovirus, RSV, and SARS-CoV-2; and seasonal coronaviruses, including 229E, HKU1, NL63, and OC43.

† Surveys were deployed using Research Electronic Data Capture via text message or email based on parent (for students) or participant (for staff members) preference. Parents and staff members were asked whether participants had respiratory virus infection signs and symptoms (i.e., fever, cough, nasal congestion, runny nose, sore throat, wheezing, shortness of breath, or none of the above) during the previous 7 days and were instructed to select all that applied.

§ 45 C.F.R. part 46.102(l)(2), 21 C.F.R. part 56; 42 U.S.C. Sect. 241(d); 5 U.S.C. Sect. 552a; 44 U.S.C. Sect. 3501 et seq.

¶ Participants enrolled were 60.7% female, 78.7% White, 6.9% Black, 7.7% multiracial, 11.3% Hispanic, 92.8% with English as their preferred language, and 31.3% qualified for free or reduced meals. School districtwide demographic characteristics for the 2022–23 school year were 51.0% female, 71.0% White, 10.4% Black, 4.5% multiracial, 10.3% Hispanic, 88.1% with English as their preferred language, and 38.0% qualified for free or reduced meals.

** Enrollment was ongoing at the time of analysis.
